# Development of a chimeric odour blend for attracting gravid malaria vectors

**DOI:** 10.1186/s12936-021-03797-w

**Published:** 2021-06-09

**Authors:** Betelehem Wondwosen, Mengistu Dawit, Yared Debebe, Habte Tekie, Sharon R. Hill, Rickard Ignell

**Affiliations:** 1 Department of Zoological Sciences, Addis Ababa University, PO Box 1176, Addis Ababa, Ethiopia; 2 Unit of Chemical Ecology, Department of Plant Protection Biology, Swedish University of Agricultural Sciences, Sundsvägen 14, PO Box 102, 230 53 Alnarp, Sweden; 3 Public Health Entomology Research Team, Ethiopian Public Health Institute, PO Box 1242, Addis Ababa, Ethiopia

**Keywords:** Chimeric, Odour, Chemical ecology, *Anopheles*, Vector, Mosquito, Control, Grass

## Abstract

**Background:**

Odour-based tools targeting gravid malaria vectors may complement existing intervention strategies. *Anopheles arabiensis* are attracted to, and stimulated to oviposit by, natural and synthetic odours of wild and domesticated grasses associated with mosquito breeding sites. While such synthetic odour lures may be used for vector control, these may have limited efficacy when placed in direct competition with the natural source. In this study, workflows developed for plant-feeding pests was used to design and evaluate a chimeric odour blend based on shared attractive compounds found in domesticated grass odours.

**Methods:**

Variants of a synthetic odour blend, composed of shared bioactive compounds previously identified in domesticated grasses, was evaluated sequentially in a two-choice olfactometer to identify a ratio-optimized attractive blend for malaria vectors. During this process, blends with ratios that were significantly more attractive than the previously identified synthetic rice blend were compared to determine which was most attractive in the two-choice olfactometer. To determine whether all volatile components of the most attractive blend were necessary for maximal attraction, subtractive assays were then conducted, in which individual components were removed for the most attractive blend, to define the final composition of the chimeric blend. Binary logistic regression models were used to determine significance in all two-choice assays. The chimeric blend was then assessed under field conditions in malaria endemic villages in Ethiopia, to assess the effect of dose, trap type, and placement relative to ground level. Field data were analyzed both descriptively and using a Welch-corrected t-test.

**Results:**

A ratio-optimized chimeric blend was identified that significantly attracted gravid *An. arabiensis* under laboratory conditions. In the field, trap captures of *An. arabiensis* and *Anopheles pharoensis* were dependent on the presence of the lure, trap type (CDC, BG Sentinel and Suna traps), placement relevant to ground level, with low release rates generally luring more mosquitoes.

**Conclusions:**

The workflow designed for the development of chimeric lures provides an innovative strategy to target odour-mediated behaviours. The chimeric lure identified here can be used in existing trapping systems, and be customized to increase sustainability, in line with goals of the Global Vector Control Response Group.

## Background

Current strategies used in the management of malaria are threatened by the development of insecticide and behavioural resistance in human malaria vectors [1, 2]. Efforts to develop tools to complement those currently in use in integrated vector management (IVM) are required, particularly those targeting exophilic mosquitoes, as an increasing proportion of people are now at risk of infective bites from mosquitoes outdoors [3–5]. To this end, it is essential to increase the understanding of the ecology and behaviour of malaria vectors outdoors, to identify novel targets for IVM tool development [5, 6]. In particular, targeting gravid malaria vectors, a life stage currently lacking tools in IVM, has the potential to reduce the mosquito density, and the vectorial capacity, of a competent mosquito population [6].

Recent research has begun to shed light on the ecology of ovipositing malaria vectors, in part, by investigating how gravid females select and discriminate among potential oviposition sites [7–12]. Aside from humidity [13], odours emanating directly from potential oviposition sites and associated vegetation are used by gravid mosquitoes for both selection of sites and stimulation of oviposition [7–11, 14–21]. Odours emanating from either wild or domesticated grasses appear to provide accurate signals for the quality of oviposition sites for *Anopheles arabiensis* and *Anopheles coluzzii*, two of the primary malaria vectors in Sub-Saharan Africa [8–11]. Through a classical chemical ecology approach, including behavioural and electrophysiological analyses of volatile compounds from the headspace volatiles of rice, maize and sugarcane, synthetic odour blends have been developed [9–11]. The behavioural response of gravid *An. arabiensis* to these blends reflects that of the natural odours [9–11]. Of the three synthetic blends, the most attractive, rice, was evaluated under semi-field conditions, demonstrating a recapture rate of more than 70% [9].

While synthetic odour lures based on natural sources may be effective, these are potentially constrained by their restricted effectiveness in direct competition with the natural odour source, and may be affected by the previous odour experience of an individual [22]. To overcome such constraints, bioactive compounds identified in preferred odour sources may be combined into a blend, thereby avoiding the direct mimicking of a natural odour [23, 24]. Such “super blends” have been used effectively to control plant pests from various insect orders [25, 26], but as of yet, are not available for the control of haematophagous insects.

In this study, the principles set out by Del Socorro et al*.* [27] and Gregg et al*.* [25] were used in designing a chimeric blend based on the previously identified attractive blends of domesticated grasses. Moreover, following the ratio-specific hypothesis proposed by Bruce et al*.* [28] and Bruce and Pickett [29], this blend was assayed for attractiveness at various ratios within that of the natural emission rates of the individual compounds under laboratory conditions. The most attractive chimeric blend was subsequently evaluated under field conditions in malaria endemic villages in Ethiopia, using different trapping methods. The findings are discussed in the context of its potential for the development of a gravid trap for malaria mosquitoes and its potential to influence the strategy and goals set by the World Health Organization through its vector ecology and management, and its sustainable development divisions [6].

## Methods

### Rearing of *Anopheles arabiensis*

*Anopheles arabiensis* (Dongola) mosquitoes were maintained at 27 ± 2 °C, 75 ± 5% relative humidity (RH) under a 12 h light: 12 h dark photoperiod. All immature stages were reared in distilled water, and the larvae allowed to feed on Tetramin^®^ fish food (Tetra, Melle, Germany), as previously described [9–11]. Adults were allowed to emerge in Bugdorm cages (30 cm × 30 cm × 30 cm; MegaView Science, Talchung, Taiwan) and supplied with sucrose solution ad libitum. Five days post-emergence (dpe), the females were offered sheep blood (Håtuna AB, Bro, Sweden) from a membrane feeder (Hemotek, Discovery Workshops, Accrington, UK) for 1 h. For the experiments, gravid (3 days post-blood meal) *An. arabiensis* were used.

### Y-tube olfactometer

The preference of gravid *An. arabiensis* to various synthetic odour blends was assessed using a Y-tube olfactometer, as previously described [8], illuminated from above with red light at 4 lx. A charcoal-filtered and humidified air stream (25 ± 2 °C, RH 65 ± 2%) flowed through the olfactometer at 30 cm s^−1^. All experiments were performed from ZT 13–17, i.e., the peak activity period of *An. arabiensis* [11]. For each experimental replicate, ten 5–7 dpe mosquitoes, with access to water but deprived of sucrose for 8 h prior to the experiment to enhance flight activity, were allowed to acclimatize for 2 h in a single cylindrical release chamber (6 cm × 10 cm inner diameter) in the experimental room prior to experiments. Ten replicates were performed for each treatment. The chamber was placed at the downwind end of the Y-tube, and females allowed 2 min to acclimatize before the door of the chamber was opened. The preference of the gravid mosquitoes was determined by counting the number of mosquitoes that entered each arm within 5 min.

For the delivery of the synthetic odour blends and the solvent control (pentane, 99.0% GC grade, Sigma, Stockholm, SE), wick dispensers, constructed from a 2 ml glass vial [9], were placed within a glass wash bottle (250 ml; Lenz Laborglas, Wertheim, Germany). Charcoal-filtered and humidified air (0.5 l min^−1^) was passed through the wash bottles and delivered via Teflon tubing into the upwind arms of the Y-tube olfactometer.

### Behavioural response to synthetic odour blends

**Fig. 1:**
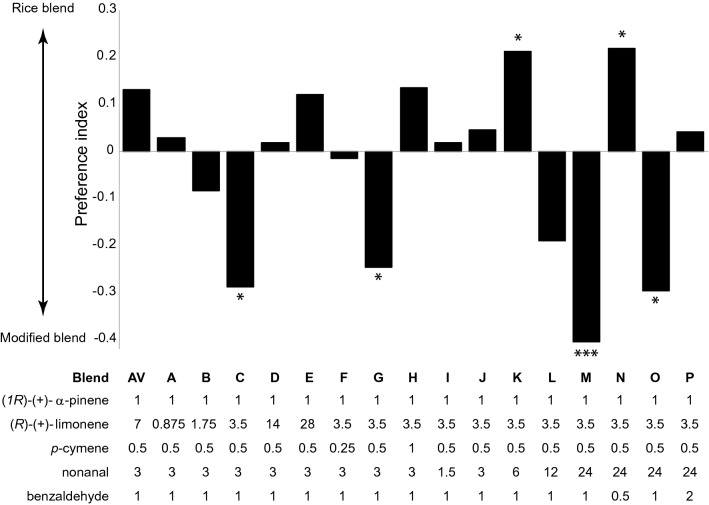
Assessment of ratio-varied blends for the attraction of gravid *Anopheles arabiensis*. Synthetic blends, composed of bioactive volatile organic compounds identified as shared among the three domesticated grass species (maize, sugarcane and rice) attractive to gravid *An. arabiensis*, are compared to the synthetic rice odour blend [9–11]. The ratio of the individual compounds was varied, as detailed in the table below the graph. The ratio of compounds in the first blend tested (AV) represents the average release rate of individual compounds among the domesticated grasses. Synthetic blends were evaluated sequentially, varying the ratio of only one compound at a time. The most preferred ratio was maintained in subsequent blends (left to right). The response variable is presented as a preference index. Asterisks indicate a significant preference for either the synthetic rice blend or the ratio-varied blends. Ten replicates, of 10 mosquitoes each, were used in each behavioural experiment

The synthetic odour blends assessed consisted of (*1R*)-(+)-α-pinene (CAS no. 7785-70-8; Sigma, 98%), nonanal (CAS no. 124-19-6; Sigma, 95%,), *p*-cymene (CAS no. 99-87-6; Aldrich, 97%), benzaldehyde (CAS no. 100-52-7; Sigma, 99%) and (*R*)-(+)-limonene (CAS no. 5989-27-5; Sigma, 97%), which are all present in the maize (*Zea mays*, variety BH660) odour blend, and shared with at least one other domesticated grass odour blend, from rice (*Oryza sativa*, variety MR3) and sugar cane (*Saccharum officinarum*, varieties Coll 48 and EAK 71-402) [9–11]. A synthetic blend, based on the average release rate of these compounds (ng min^−1^), blend AV, was initially constructed containing (*1R*)-(+)-α-pinene, (*R*)-(+)-limonene, *p*-cymene, nonanal and benzaldehyde at a ratio of 1:7:0.5:3:1. Four behavioural experiments were designed to identify a ratio-optimized blend containing only those components that contribute to the bioactivity of the blend. (1) The behavioural response of gravid *An. arabiensis* to blend AV was assessed against the most attractive domesticated grass synthetic blend, rice [9, 11], in the Y-tube olfactometer demonstrating no significant difference. (2) Subsequently, the ratio of individual components in blend AV was altered, generating blends A–P (Fig. 1). These blends (A–P) were assessed against the synthetic rice odour blend to identify ratio-optimized blends, which were superior in attracting gravid *An. arabiensis*. (3) This resulted in four blends which were then compared serially in pairwise comparisons, in which each subsequent comparison included the most attractive blend from the previous comparison. (4) The most highly preferred synthetic blend of these four (blend M) was then used in subtractive assays in which individual compounds were removed from the full synthetic blend, and tested against the full synthetic blend.

Behavioural preference was assessed using a preference index (PI): PI = (T_1_ − T_2_)/(T_1_ + T_2_), where T_1_ is the number of mosquitoes associated with test odour 1 and T_2_ the number of mosquitoes associated with test odour 2. The data was analysed using binary logistic regression in SPSS Statistics for Windows, (v 20, Armonk, NY: IBM Corp), in which choice was the dependent variable weighted by the number of mosquitoes used in the assay.

### Assessment of chimeric blend under field conditions

The chimeric blend was assessed under field conditions in malaria endemic villages, located nearby the town of Meki in the Oromia region (8° 11′ 08ʺ N, 38° 81′ 70 ʺ E) and near a village called Sile (5° 53′ 24ʺ N, 37° 29′ 24ʺ E) in Arba Minch Zuria district of the Gamo Gofa zone in Ethiopia. A detailed description of the study villages is outlined by Carter et al*.* [30] and Debebe et al*.* [31]. Field experiments around Meki were conducted following the long rainy season (September–October 2017) using BG-Sentinel traps (BioGents AG, Regensburg, DE; [32]) and CDC light traps (BioQuip Products Inc, CA, US). The BG-Sentinel traps were placed on the ground, with (4.5 l; BG-W) and without (BG) water, while CDC light traps were placed at 30 cm (CDC-L) or 100 cm (CDC-H) above ground. Five traps of the same type were set in a block and separated by ca. 20 m along a tangent, set in areas shaded by vegetation (50–70 m from households), which were previously identified to be optimal resting sites for *An. arabiensis* [31]. In each of four blocks, each trap was baited with one of four doses of the chimeric blend or a control (see below). The location of the treatments and control were then rotated for five nights, so that each treatment and control visited each location once. Thereafter, the trap types were exchanged among the blocks in a randomized block design for a total of 5 replicates, resulting in a total of 20 trap nights per treatment and trap type. Each block was separated from the others by up to 500 m. To monitor the mosquito population density in the area, CDC light traps were set each week, indoors and outdoors, 1 to 2 km from the locations of the gravid traps, so as not to interfere with the local study area populations.

In Sile, a different study design was used. A total of 12 houses with similar construction characteristics were selected, and one Suna trap (BioGents AG; [33]) per house was suspended in the shade next to the house under the eaves at 30 cm above the ground and away from windows and doors. Treatments and control were assigned to each house, at the start of each experiment and then rotated following a Latin square design with a 1 × 11 rectangular distribution, for a total of 11 nights (132 trap nights). Experiments were carried out around Sile during the long rainy season (May–June 2019).

In both sites, the chimeric blend was released using wick dispensers with heptane as the solvent to ensure constant release of the odour blend throughout the 12 h experiment. These dispensers were introduced in each trap type at 18:00 ± 1 h, at which time the traps were connected to the 12 V batteries. The dispensers were removed at 8:00 the next morning, when the traps were emptied. Experiments conducted near to Meki were designed to assess dose-dependent attraction and capture of mosquitoes in which four doses of the blend, presented in half decadic steps (3–100 ng µl^−1^), were compared to a control (heptane). In Sile, experiments were conducted using a single dose (3 ng µl^−1^), which was tested against a control (heptane). Comparison of the mean number of mosquitoes in the control and odour-baited traps was made by using Welch-corrected t-test accounting for unequal variances and sample size in SPSS (v 25, Armonk, NY: IBM Corp). For both sites, the number of captured mosquitoes per trap and treatment were recorded, and the mosquitoes were placed on silica gel before being transported to the laboratory for species, sex and physiological state identification [34, 35].

## Results

### Development of a chimeric odour blend for gravid mosquitoes

**Fig. 2:**
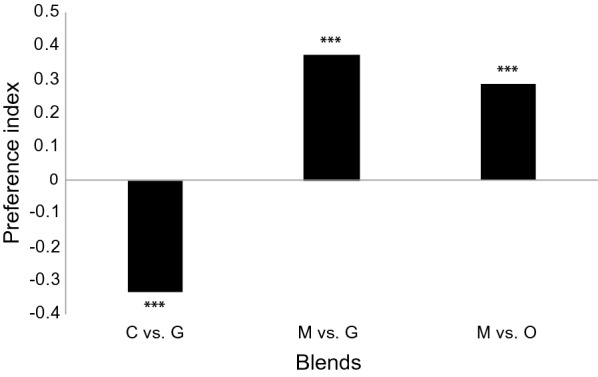
Development of a chimeric odour blend for the attraction of gravid *Anopheles arabiensis*. Synthetic blends, composed of various ratios of bioactive compounds identified in Fig. 1, were assessed in a two-choice assay demonstrating a preference for blend M. The response variable is presented as a preference index. Asterisks indicate a significant preference for either blend. Ten replicates, of 10 mosquitoes each, were used in each behavioural experiment

**Fig. 3:**
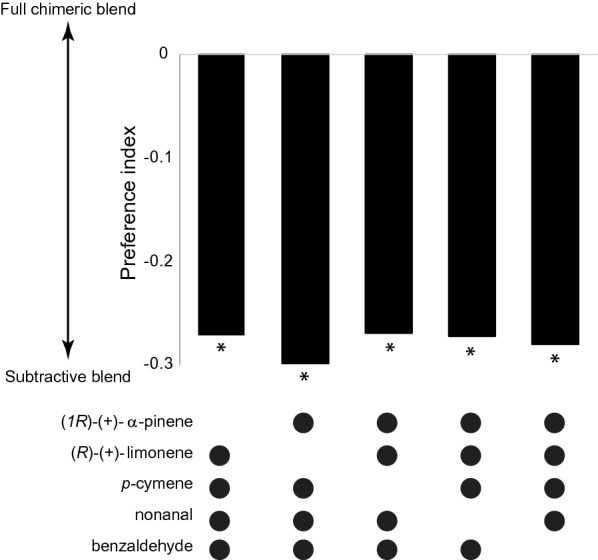
Attraction of gravid *Anopheles arabiensis* to the chimeric odour blend requires all components. The subtraction of individual components from the full chimeric blend significantly reduces the attraction of gravid females compared to the full chimeric blend. The response variable is presented as a preference index. Asterisks indicate a significant preference for either blend. Ten replicates, of 10 mosquitoes each, were used in each behavioural experiment

The release rate of the individual compounds selected for the development of the chimeric blend vary within the natural emanations of the domesticated grasses [9–11]. The average release rate of these compounds was used to construct a basic blend (AV) from which subsequent blends were modified in order to identify the optimal ratio among the active components (Fig. 1). Gravid mosquitoes preferred the synthetic rice odour blend and the AV blend equally (Fig. 1). The ratio of individual compounds in subsequent modified blends were varied while maintaining the release rate of (*1R*)-(+)-α-pinene constant. These blends were tested sequentially, where the optimal ratio of a given compound, starting randomly with (*R*)-(+)-limonene, was carried over to the evaluation of the next series of modified blends (Fig. 1; blends A to P). The modified blends were compared against the synthetic rice odour blend, in which blends C (*χ*^2^ = 5.839, 95% CI 0.052–0.735; P < 0.016), G (*χ*^2^ = 5.505, 95% CI 1.345–27.231; P < 0.019), M (*χ*^2^ = 9.525, 95% CI 1.094–1.714; P < 0.0001) and O (*χ*^2^ = 5.456, 95% CI 1.351–31.175; P < 0.020) were significantly preferred by gravid *An. arabiensis* (Fig. 1). To assess which of these modified blends were preferred by gravid mosquitoes, pairwise comparisons were made revealing that blend M was the most attractive blend (blend C vs. G, *χ*^2^ = 7.356, 95% CI 1.061–1.910, P < 0.0001; blend M vs. G, *χ*^2^ = 9.492, 95% CI 1.161–1.821, P < 0.0001; and blend M vs. O, *χ*^2^ = 6.189, 95% CI 0.925–1.875, P < 0.0001; Fig. 2). Subtractive blends were then assessed demonstrating that the removal of individual components from blend M significantly reduced attraction (χ^2^ = 7.175, 95% CI 1.239–1.874, P < 0.0001; Fig. 3).

### Evaluation of the chimeric blend under field conditions

**Fig. 4:**
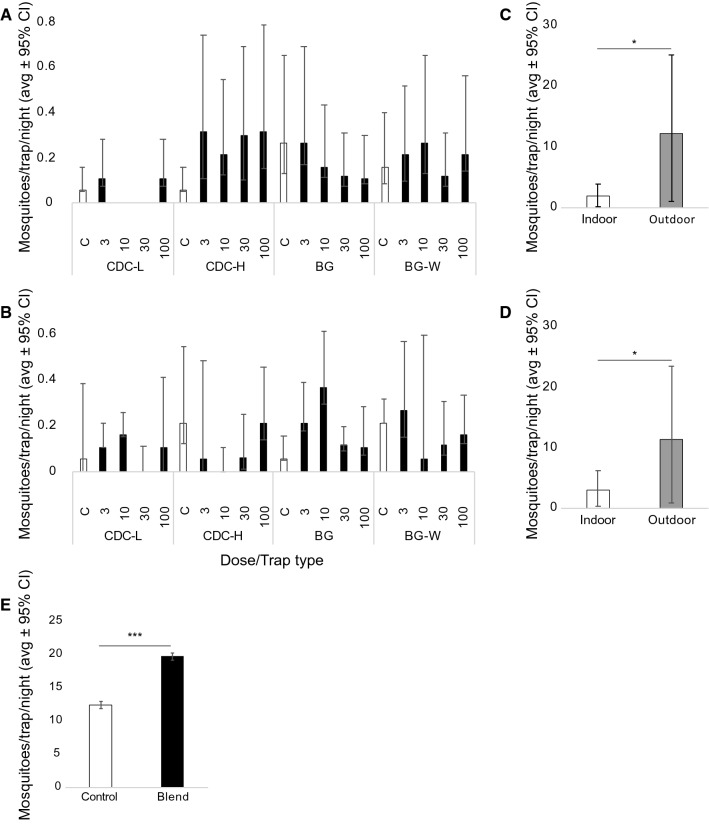
Assessment of the chimeric odour blend under field conditions. In Meki, *Anopheles arabiensis* (**A**) and *An. pharoensis* (**B**) were caught using CDC traps hung either low (CDC-L) or high (CDC-H) and BG-Sentinel traps, with (BG-W) and without water (BG). Traps were baited with a solvent control (**C**) or different doses of the chimeric blend. Control traps set indoors and outdoors at a nearby site demonstrated a low population density of both *An. arabiensis* (**C**) and *An. pharoensis* (**D**) in the area, with CDC traps catching more *Anopheles* mosquitoes outdoors near the houses. In Sile, Suna traps, set next to houses, baited with the chimeric blend (3 ng µl^−1^) caught a significantly higher number of *An. arabiensis* than unbaited traps (**E**)

In the Meki district, the chimeric blend was evaluated to identify the optimal release rate, trap type and trap height for luring *Anopheles* mosquitoes under field conditions, with traps placed in shaded areas, 50–70 m from households. A total of 2612 mosquitoes were collected in 336 trap nights (experiment: 16 traps per night for 19 nights, one night removed due to severe rain; control: 16 traps over 2 nights), of which 79.2% were *Culex* species. *Anopheles arabiensis*, representing 9.2% of the total catch*,* is the only species within the *Anopheles gambiae* species complex reported within the area [36]. The remaining species caught were *Anopheles pharoensis* and *Anopheles ziemanni*, representing 9.5% and 2.1%, respectively. Mosquitoes caught during experimental nights were assessed for sex and physiological state (Table 1). The four trap types performed differently under field conditions (Fig. 4A, B). The height of the CDC traps affected the number of *An. arabiensis* caught, with traps placed close to the ground capturing up to 6 times more mosquitoes per trap per night than the control, irrespective of the chimeric blend release rate (blend M) (Fig. 4A). Moreover, the presence or absence of water in BG-Sentinel traps inversely affected the number of *An. arabiensis* caught per trap per night (Fig. 4A). While captures of *An. pharoensis* in CDC traps were highly stochastic, females of this species were caught in a dose-dependent manner in BG-Sentinel traps, both with and without water, with up to 8 times more females caught per trap per night in the presence of the chimeric blend than the control (Fig. 4B).

**Table 1:** Species, sex and physiological state of mosquitoes caught during experimental nights in the two study sites

	Female				Male
	Unfed	Fed	Semi-Gravid	Gravid	
Meki					
* Anopheles arabiensis*	39	4	2	8	10
* Anopheles pharoensis*	22	1	2	5	21
* Anopheles ziemanni*	3	0	0	0	0
*Culex* spp.	1034	20	65	291	263
Sile					
* Anopheles arabiensis*	560	39	1	0	0
* Anopheles pharoensis*	4	0	0	0	0
* Anopheles ziemanni*	0	1	0	0	0
* Culex* spp.	nd	nd	nd	nd	nd

*nd* not done

Control collections of mosquitoes made indoors and outdoors in Sile during the course of the experiments with CDC light traps demonstrated that the density of vectors in the study area was relatively low during the time of the experiments. In indoor collections, a mean of 1.8 (95% CI 1.6–2.0) and 2.9 (95% CI 2.5–3.3) mosquitoes per trap per night was found for *An. arabiensis* and *An. pharoensis*, respectively. Outdoor collections near the houses, on the other hand, demonstrated a mean of 12.1 (95% CI 11.2–13.1) and 11.3 (95% CI 10.3–12.2) mosquitoes per trap per night for *An. arabiensis* and *An. pharoensis*, respectively.

In Sile, 2492 *Anopheles* were collected in Suna traps placed outdoors of houses over 11 nights, of which 99.4% were *An. arabiensis*, the only member of the *An. gambiae* species complex in the study area [37, 38]. The remaining *Anopheles* species collected were *An. pharoensis* and *An. ziemanni*, representing 0.6% of the total number of *Anopheles* mosquitoes caught. Traps baited with the chimeric blend (3 ng µl^−1^) caught significantly higher numbers of *An. arabiensis* per trap per night than the controls (Welch-corrected t = 2.1, P < 0.0001; Fig. 4E).

## Discussion

Odour-based tools, targeting gravid *An. arabiensis*, are required to complement existing intervention strategies, which mainly target the indoor feeding and resting population. Gravid *An. arabiensis* are attracted to natural and synthetic odours of domesticated grasses [9–11, 39]. Bioactive volatile compounds identified and shared among these domesticated grasses were used to develop a ratio-optimized chimeric odour blend, providing a workflow for the development of blends that target oviposition-site seeking mosquitoes. In a two-choice assay, gravid mosquitoes preferred the chimeric odour blend (blend M) over that of a synthetic odour of rice, previously demonstrated to be highly effective in attracting and capturing mosquitoes in laboratory and semi-field experiments [9]. While field assessment demonstrated that the chimeric blend (blend M) may be effective in direct competition with natural odour sources, future work is required to enhance the applicability of this odour-based intervention tool.

Originally described by Del Soccorro et al*.* [27], synthetic attractant blends do not need to rely on a mimic of bioactive compounds in a single resource. In fact, blends of bioactive compounds that are shared among attractive resources may provide an enhancement of attraction, thus providing a competitive advantage compared to any existing natural source [26, 27, 39]. Similar to Gregg et al*.* [25, 40], we used an empirical approach to develop the chimeric blend used to attract gravid *An. arabiensis*, in which the ratio of the individual bioactive compounds was optimized [28, 29]. While the inclusion of additional bioactive compounds from preferred, and even non-preferred, vegetation associated with mosquito potential breeding sites may improve the efficacy of the chimeric blend under field conditions, the results from the laboratory bioassays clearly demonstrate that the chimeric blend may be superior to that of previously identified attractants for gravid *Anopheles* mosquitoes [7, 9–11, 15–17, 21]. Moreover, this study provides proof-of-principle that chimeric blends, also referred to as super-blends, may be developed for surveillance and control of vector mosquitoes, by combining similar approaches as those used for plant-feeding insect pests. With recent progress in understanding the chemical ecology of behaviours involved in floral, host and oviposition-site selection, it is becoming clear that bioactive compounds are used by mosquitoes parsimoniously [12, 24]. These compounds provide a basis for the future development of chimeric blends that attract mosquitoes of different species and physiological states.

The number of mosquitoes caught in the field experiments was dependent on trap type and placement with respect to ground level. Similar to that reported by Lindh et al*.* [7], the number of mosquitoes caught per trap per night was low, which can be explained, at least in part, by the low population densities at the time of study. By placing the traps in hotspots for resting mosquitoes [31], shaded sites that were ≥ 50 m from the household, a higher number of mosquitoes were caught, than those reported by Lindh et al*.* [7], even after adjusting for differences in population density. However, trap capture was still low, possibly reflecting the difficulty in luring gravid mosquitoes from their resting sites, and the current understanding of how gravid mosquitoes move within the landscape to locate potential oviposition sites. These field experiments designed to assess the dose-dependent attraction of malaria vectors, and generally identified the lower range of doses (3–10 ng µl^−1^) to be the most effective. This is consistent with our previous results from semi-field trials with the synthetic rice blend, emphasizing the superior sensitivity of the olfactory system of *Anopheles* mosquitoes [9]. The high density of active mosquitoes near households led us to evaluate a low release rate of the chimeric blend outdoors and next to the houses, which resulted in significantly higher numbers of captures in traps baited with the lure. Whether distance from the households affects the efficacy of the lure described in this study needs further analysis, as this was not directly assessed here. The data presented in this study suggest that the activity of *Anopheles* mosquitoes with various physiological states varies depending on distance from the households, with gravid mosquitoes being more amenable to trapping close to rest sites ≥ 50 m from households, which is in line with previous observations [31].

From this study and others [7, 41, 42], it is obvious that trap type and placement with respect to ground level is critical for ensuring the optimal efficacy of odour-based lures for malaria vectors. Previous evaluations of gravid traps targeting gravid *Anopheles* mosquitoes have identified the presence of water in, the direction of airflow into (up- or down-draft) and the placement of the trap with respect to ground level to be important factors when capturing these females [7, 9, 41, 42]. In addition, trap type has been identified as a critical factor when capturing host-seeking *Anopheles* mosquitoes, with various versions of the BG-Sentinel traps, including the BG-Malaria trap and the Suna trap, often demonstrated to be superior compared to other trap types, e.g., the CDC light trap [33, 43, 44]. Using a similar approach to that of Batista et al. [44], 3-dimensional video-graphic analysis may be used to improve the design of gravid traps for *Anopheles* mosquitoes. The addition of other cues, including water vapor [13] and visual stimuli [41, 45], should be considered in future development of trapping systems for gravid *Anopheles*.

## Conclusions

The Global Vector Control Response (WHO 2017–2030) lists the innovation of new tools, particularly those targeting vectors outdoors, with the express purpose to integrate these in sustainable IVM programs as one of two foundational elements for effective and locally-adaptive vector control systems. Innovations, based on the fundamental understanding of behaviours affecting vectorial capacity, e.g., oviposition, are critical to tackle the increased population of outdoor malaria vectors. The identification of a chimeric odour blend has yielded a workflow designed for the development of odour-based lures based on the natural odour space of vector insects, here resulting in a lure for gravid malaria vectors. This lure may be used in existing trapping systems, or may serve as the basis for the development of novel systems, further designed to optimize trap capture of *Anopheles* mosquitoes. Chimeric lure systems can be customized to the local vector environments with minimal input, once local ecological conditions are known, and with multiple lures available, these may be used in rotation to increase sustainability by avoiding behavioural resistance to any one blend.

## Data Availability

All data generated or analysed during this study are included in this published article.

## Abbreviations

AV: Average blend
BG: Biogents
CAS no.: Chemical abstracts service number
CDC: Centers for disease control and prevention
CI: Confidence interval
